# Supplementary material for: Unconventional interfacial water structure of highly concentrated aqueous electrolytes at negative electrode polarizations
Source: Nat Commun. 2022 Sep 10;13:5330. doi: 10.1038/s41467-022-33129-8 (PMC9464189; doi:10.1038/s41467-022-33129-8)
Supplement: Supplementary file 1 — Supplementary Information [file 41467_2022_33129_MOESM1_ESM.pdf]

Supplementary Information

Unconventional interfacial water structure of highly concentrated aqueous electrolytes at negative electrode polarizations

Chao-Yu Li[1,5], Ming Chen[2,5], Shuai Liu[3], Xinyao Lu[4], Jinhui Meng[1], Jiawei Yan[3], Héctor D. Abruña[4], Guang Feng[2*], Tianquan Lian[1*]

[1]Department of Chemistry, Emory University, Atlanta, Georgia 30322, United States.

[2]State Key Laboratory of Coal Combustion, School of Energy and Power Engineering, Huazhong University of Science and Technology (HUST), Wuhan, 430074, China.

[3]State Key Laboratory of Physical Chemistry of Solid Surfaces, College of Chemistry and Chemical Engineering, Xiamen University, Xiamen 361005, China.

[4]Department of Chemistry and Chemical Biology, Cornell University, Ithaca, New York 14853, United States.

[5]These authors contributed equally to this work.

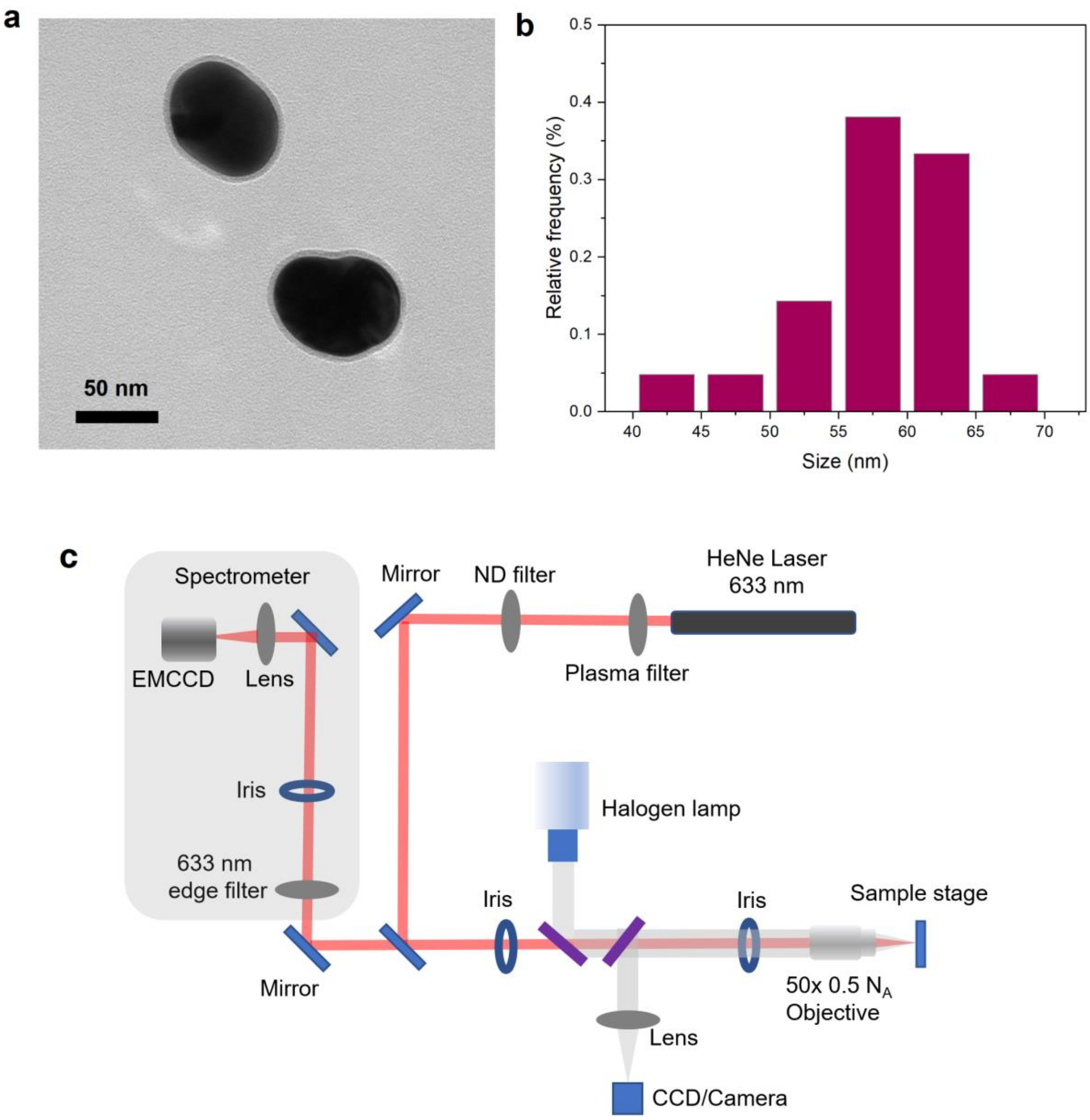

Supplementary Fig. 1 | a, TEM image of the shell-isolated Au nanoparticles consisting of a Au core of average diameter of ~57 nm and a SiO_2 shell of ~2 nm. **b**, Histogram of Au sizes. **c**, Schematic of home-build setup for *in situ* Raman spectroscopic measurement.

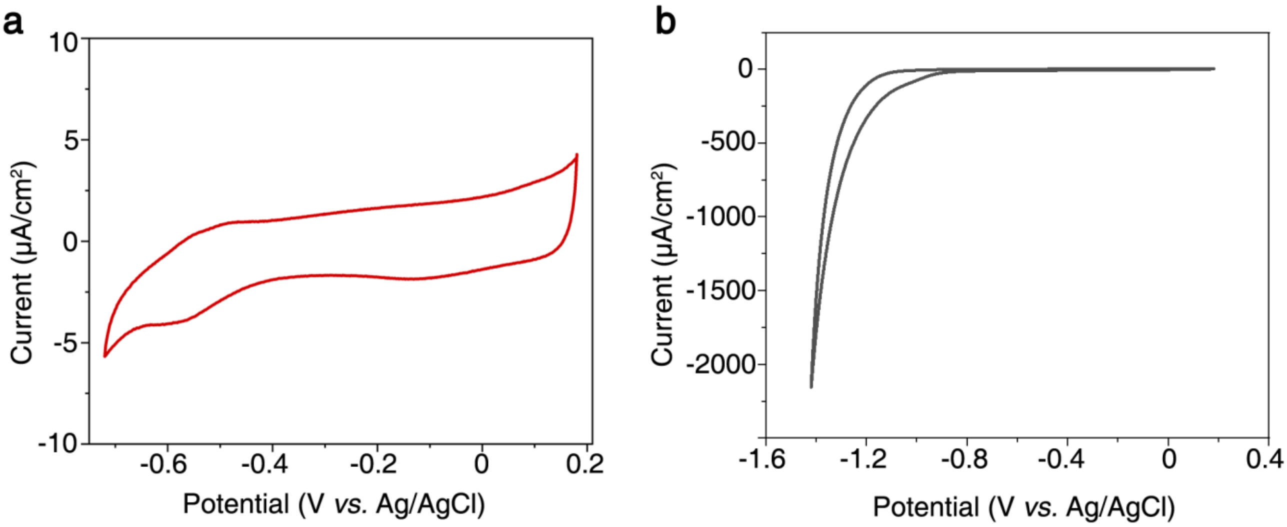

Supplementary Fig. 2 | Cyclic voltammogram of Au(111) in 21 m LiTFSI electrolyte within the different potential ranges. In both (**a**) and (**b**), the scan rate is 10 mV/s.

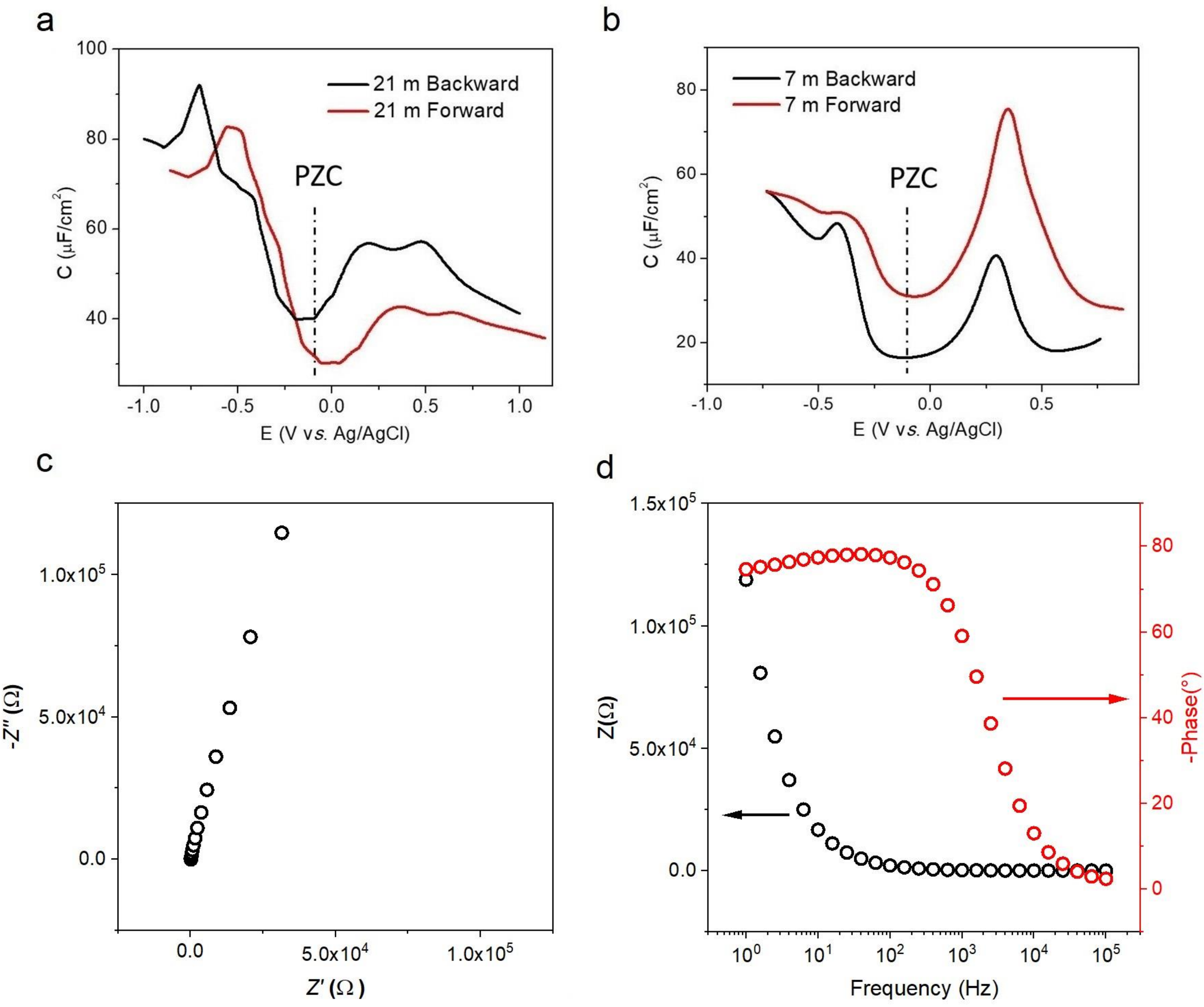

Supplementary Fig. 3 | a and **b**, Potential of zero charge (PZC) measurements of Au(111) in 21 m (**a**) and 7 m (**b**) LiTFSI aqueous electrolytes. The dashed line denotes the position of the PZC. **c** and **d**, Nyquist (**c**) and Bode (**d**) plots of the complex impedance of Au(111) in 21 m LiTFSI aqueous electrolyte at 0 V vs. Ag. **Z** is the modulus of the impedance, **Z'** and **Z''** is the real and imaginary part of the complex impedance, respectively.

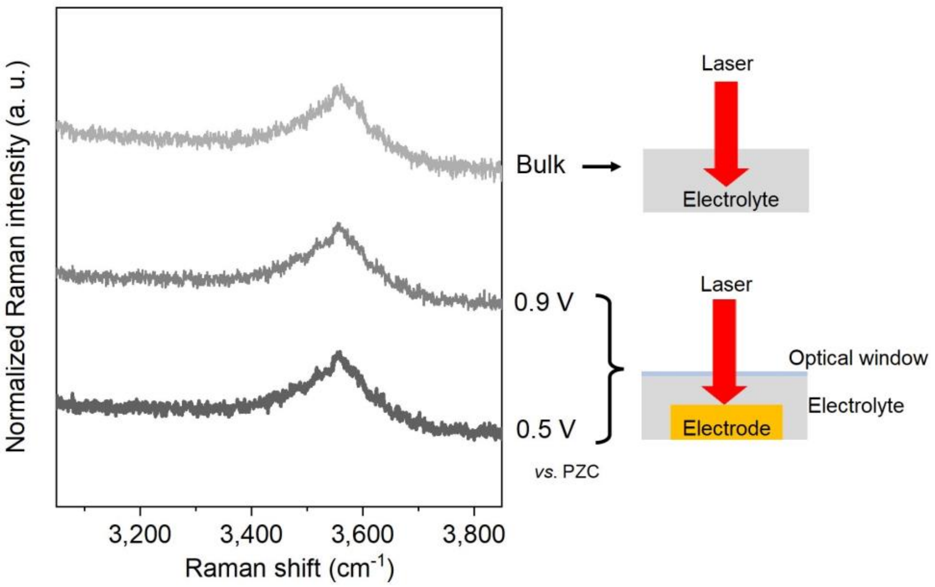

Supplementary Fig. 4 | Raman spectra of the OH stretching mode at a Au(111) surface measured in 21 m LiTFSI aqueous electrolyte at 0.5 and 0.9 V, respectively. Raman spectrum from bulk phase is presented for comparison. The corresponding schematics for Raman spectroscopic measurements of bulk phase and interfical region are shown in the right.

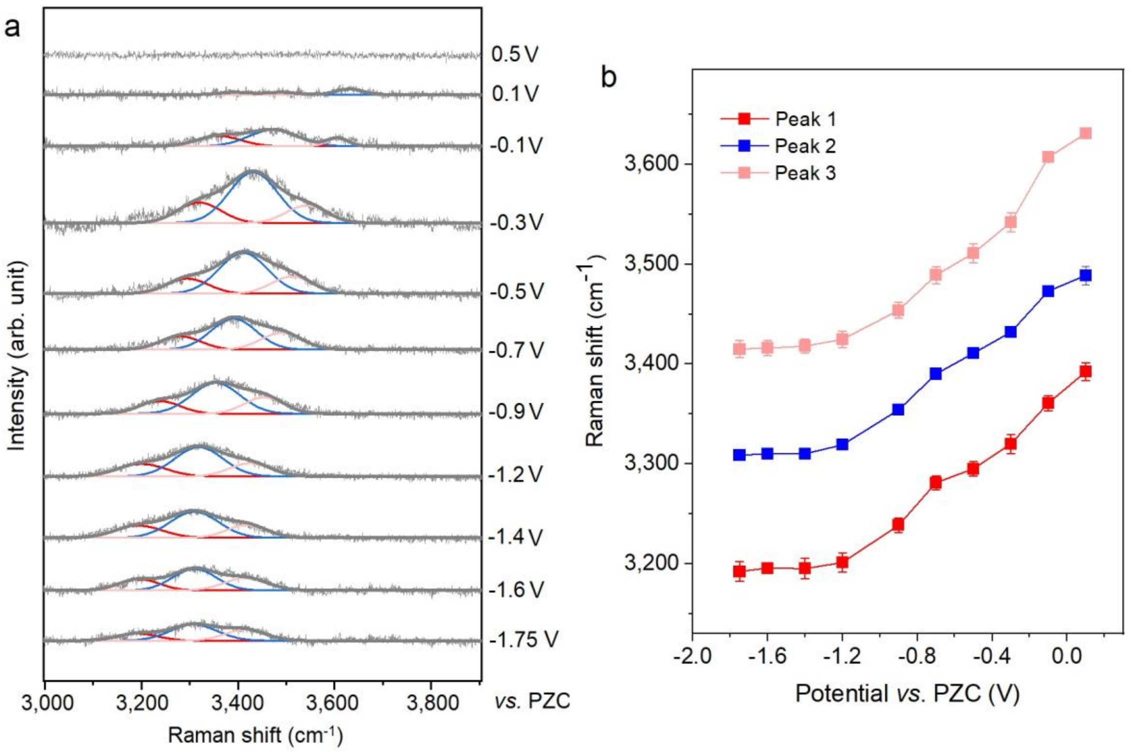

Supplementary Fig. 5 | a, *In situ* electrochemical Raman spectra of OH stretching mode at a Au(111) surface measured in 7 m aqueous LiTFSI electrolyte (grey line) and their fit to the sum of three Gaussian bands. **b**, The corresponding potential-dependent Raman shifts of three Gaussian peaks obtained from the fits shown in **a**. The error bars present the standard error.

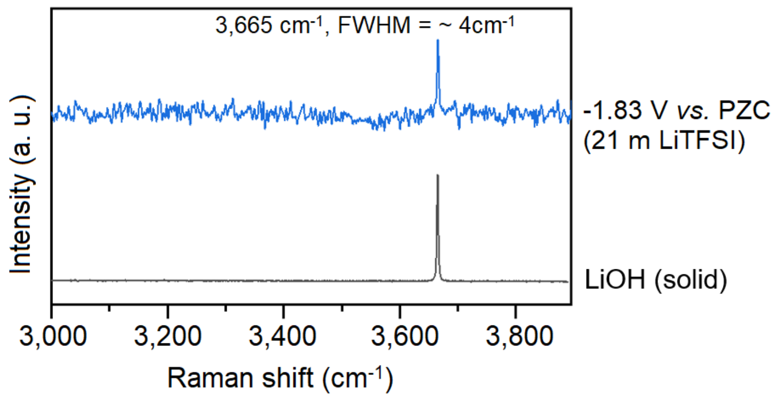

Supplementary Fig. 6 | *In situ* Raman spectrum obtained at -1.83 V in 21 m LiTFSI aqueous electrolyte and Raman spectrum of LiOH solid sample.

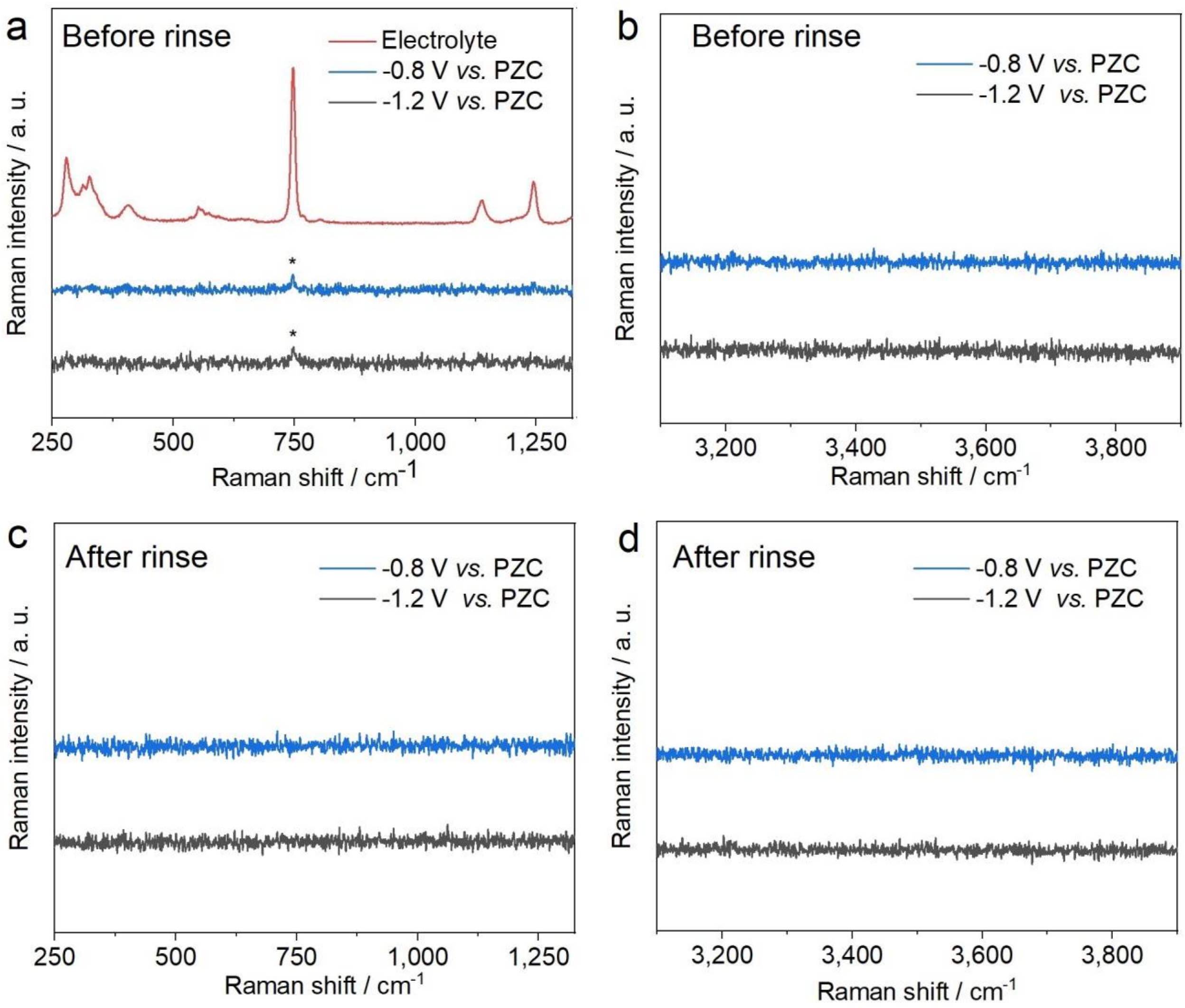

Supplementary Fig. 7 | *Ex situ* Raman spectra of electrode surface after holding the potentials at -0.8 V for 24 hours (blue) and -1.2 V for 30 min (black), respectively. Raman spectrum of 21 m WiS electrolyte is presented in **a** for comparison. In **a** and **b**, the Raman spectra were obtained without rinsing the electrode surface, while in **c** and **d**, the same electrode surface was rinsed with ultra-pure water and then dried in Argon at 25°C. The peak marked by asterisk in **a** is from the residual electrolyte (S-N-S bending mode of LiTFSI) on the surface.

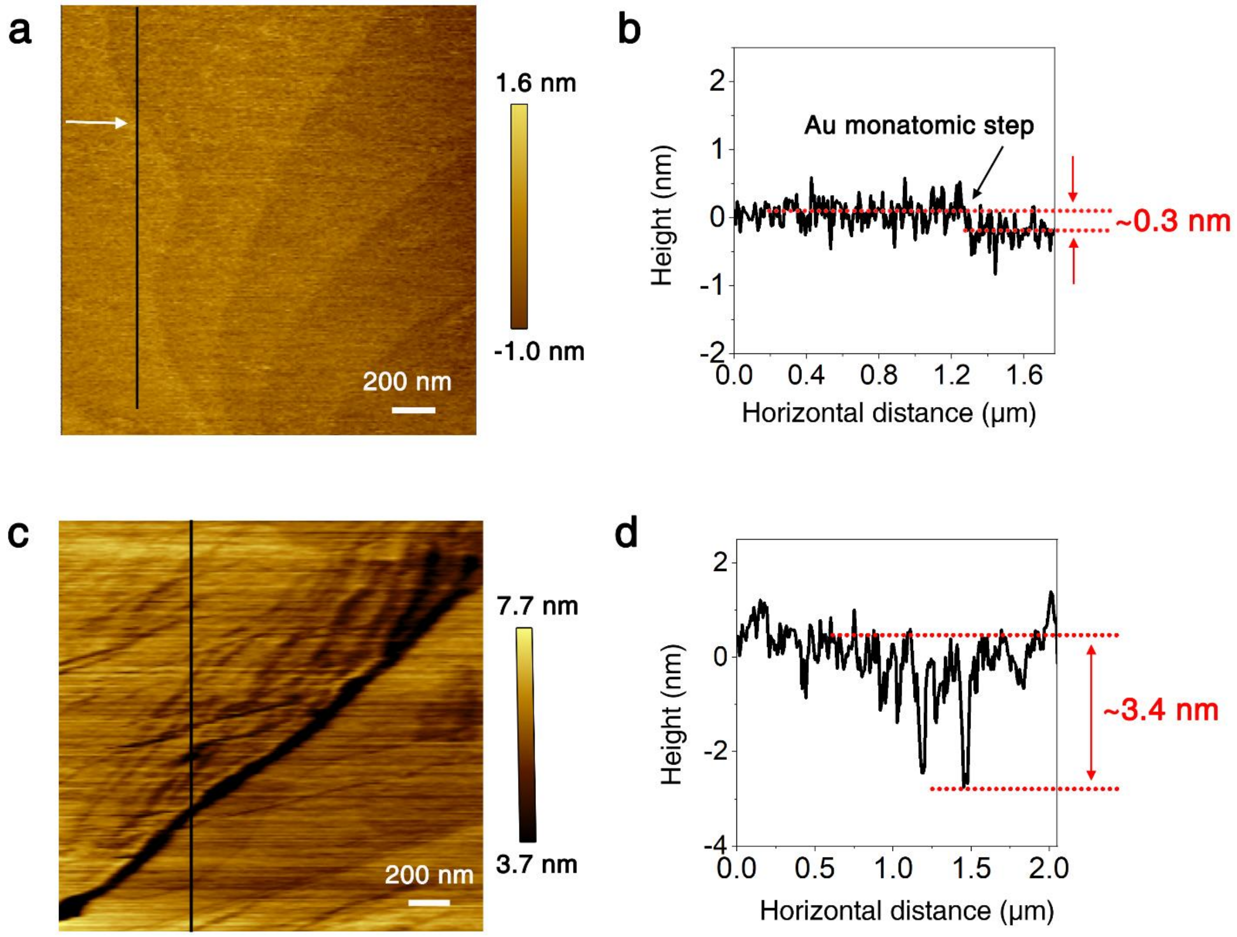

Supplementary Fig. 8 | (**a** and **c**) AFM images of Au(111) electrode surface after holding in 21 m LiTFSI aqueous electrolyte at ~ -1.2 V (**a**) and ~ -1.8 V (**c**) for 5 min, respectively. (**b** and **d**) The height profiles of the Au(111) electrode surface corresponding to the black lines in **a** (**b**) and **c** (**d**), respectively**.** The white arrow in **a** and the black arrow in **b** remark the Au monatomic step. The fluctuation of the background in (**b**) is due to the noise from the instrument.

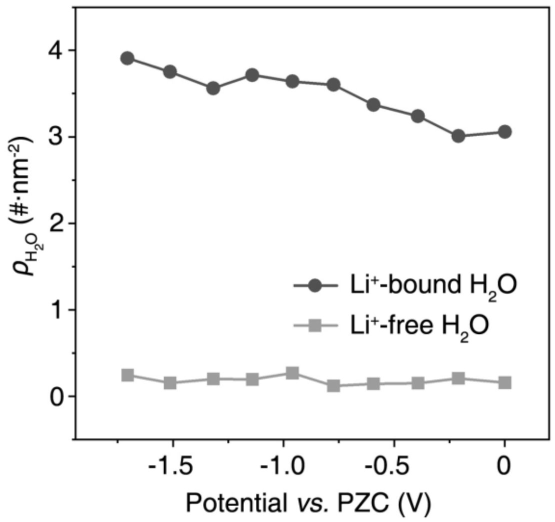

Supplementary Fig. 9 | Simulated accumulative number densities of Li^+-bound water (black line) and Li^+-free water (gray line) in the interfacial region in 21 m LiTFSI aqueous electrolyte. The accumulative number density is defined as the cumulated number of Li^+-bound water per unit area in the interfacial region, where the interfacial region is the first adsorbed layer (0-0.4 nm for Li^+-bound water).

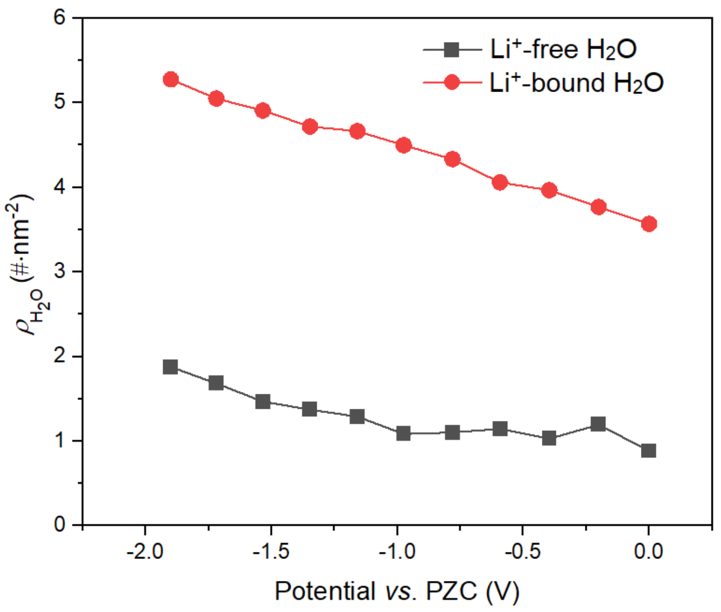

Supplementary Fig. 10 | Simulated accumulative number densities of Li^+-bound water (red line) and Li^+-free water (black line) in the interfacial region in 7 m LiTFSI aqueous electrolyte. The accumulative number density is defined as the cumulated number of Li^+-bound water per unit area in the interfacial region, where the interfacial region is the first adsorbed layer (0-0.4 nm for Li^+-bound water).

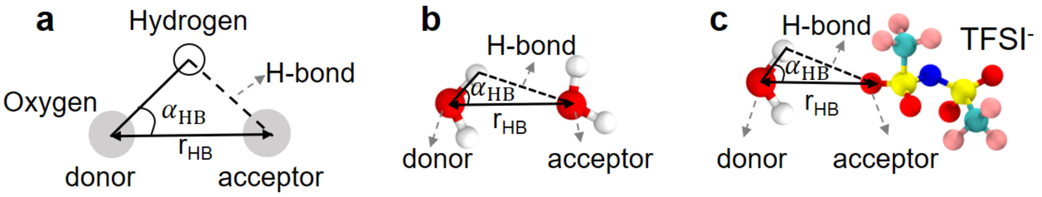

Supplementary Fig. 11 | a, Schematic of geometrical criterion for the H-bond (HB): hydrogen bond length (r_{HB}) and the angle (α_{HB}) between OO and OH. **b-c**, The schematic H-bond between H_2O and H_2O (**b**) and $TFSI^-$ (**c**). In the formation of a H-bond with a water molecule as a donor, another water molecule (**b**) or $TFSI^-$ (**c**) can act as an acceptor, where the dash line denotes the H-bond.

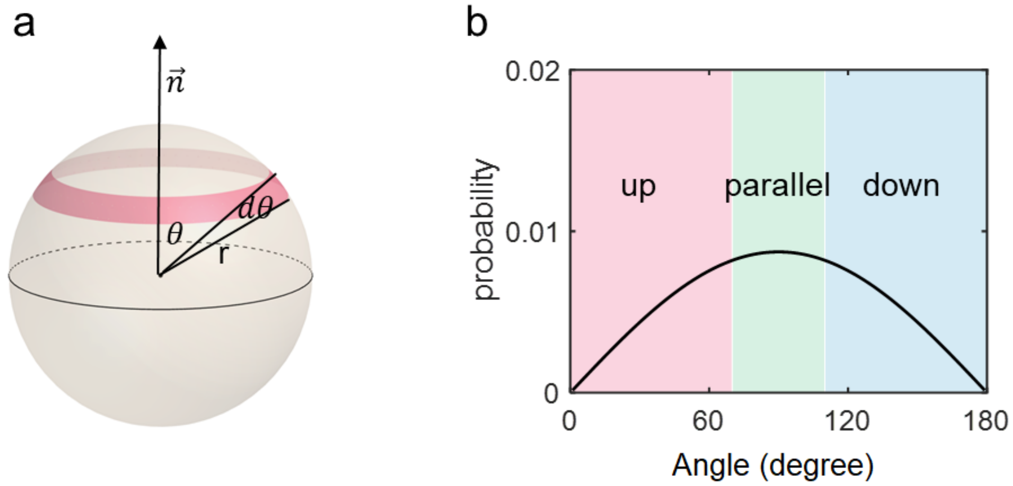

Supplementary Fig. 12 | a. Schematic for the solid angle. **b.** Angular distribution for a homogeneous system. The angle θ_{OH} can be classified into three regions: 0~70º, 70~110º, and 110~180º for 'H-up', parallel, and 'H-down' OH bond, respectively.

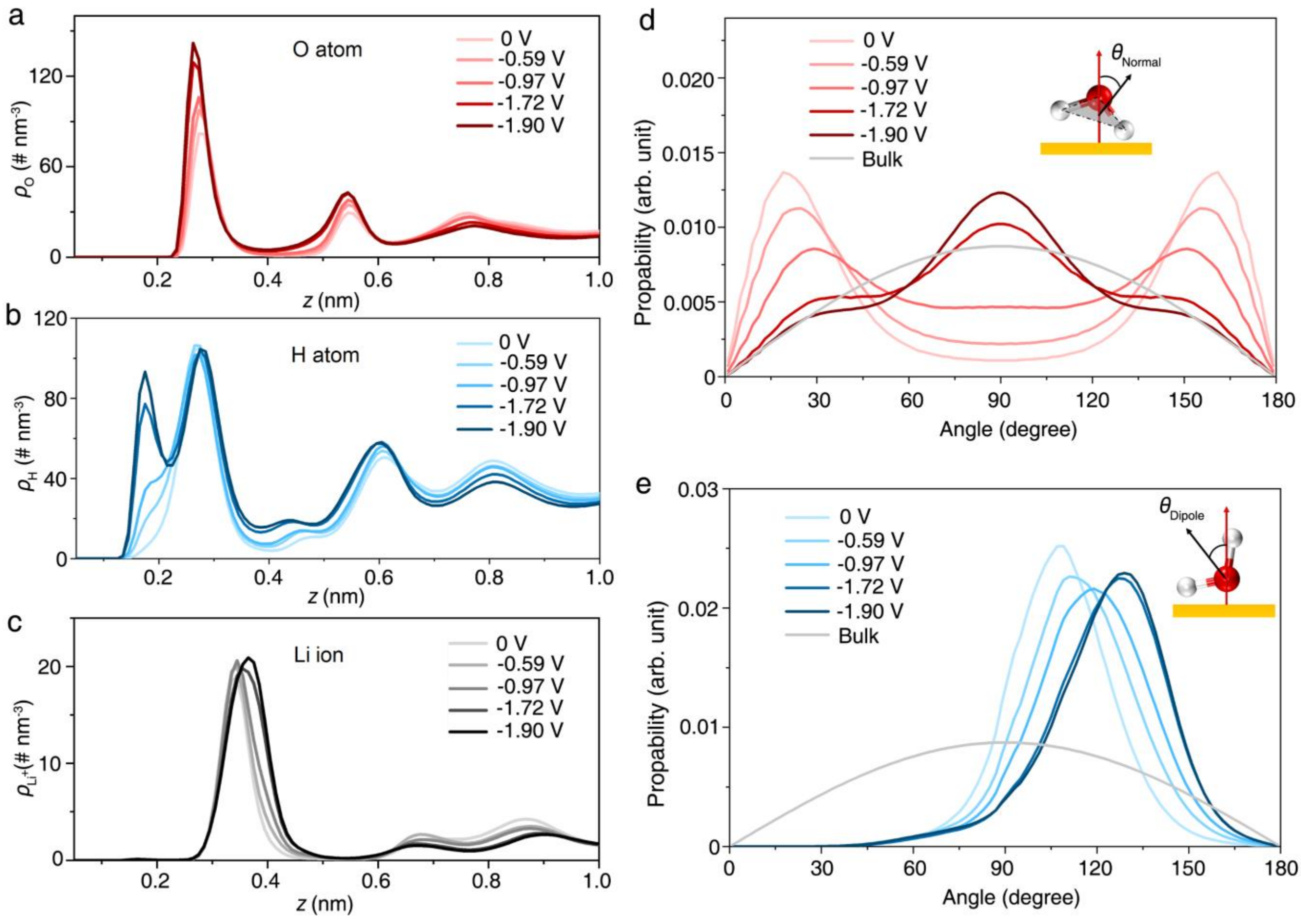

Supplementary Fig. 13 | a-c, Number densities (ρ) of O atom (**a**) and H atom (**b**), and Li^+ (**c**) in 7 m LiTFSI aqueous electrolyte. **d-e**, The normal orientation (**d**) and dipole orientation (**e**) of interfacial water in 7 m LiTFSI aqueous electrolyte.

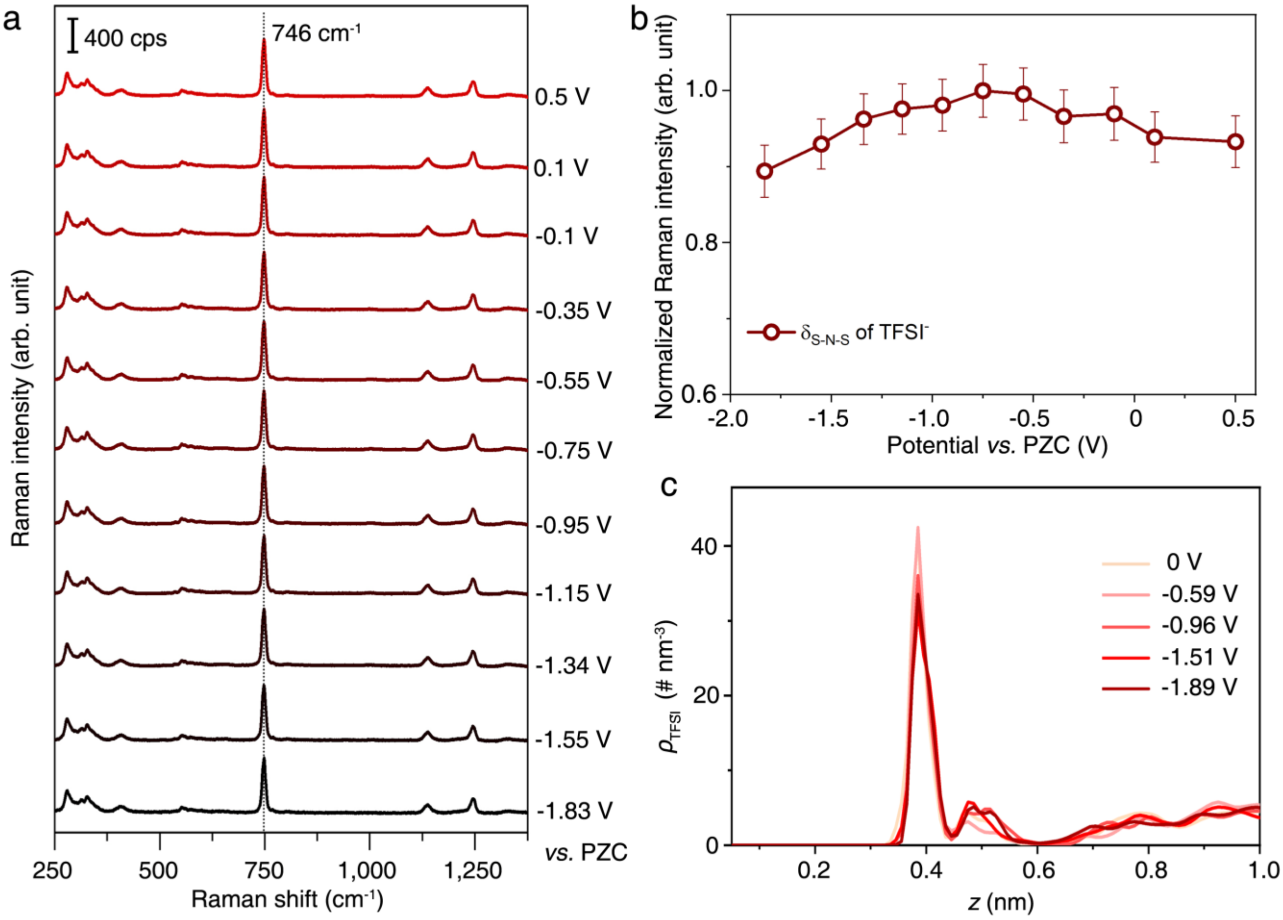

Supplementary Fig. 14 | a, *In situ* electrochemical Raman spectra of the interfacial $TFSI^-$ at indicated potentials from +0.5 V to -1.83V in 21 m LiTFSI aqueous electrolyte. **b**, Normalized Raman intensities of $\delta_{S\text{-}N\text{-}S}$ band of $TFSI^-$ as a function of potential. The error bars present the standard error. **c**, Simulated number density of $TFSI^-$ (center of mass) as a function of distance from the electrode surface at indicated potentials in 21 m LiTFSI electrolyte.

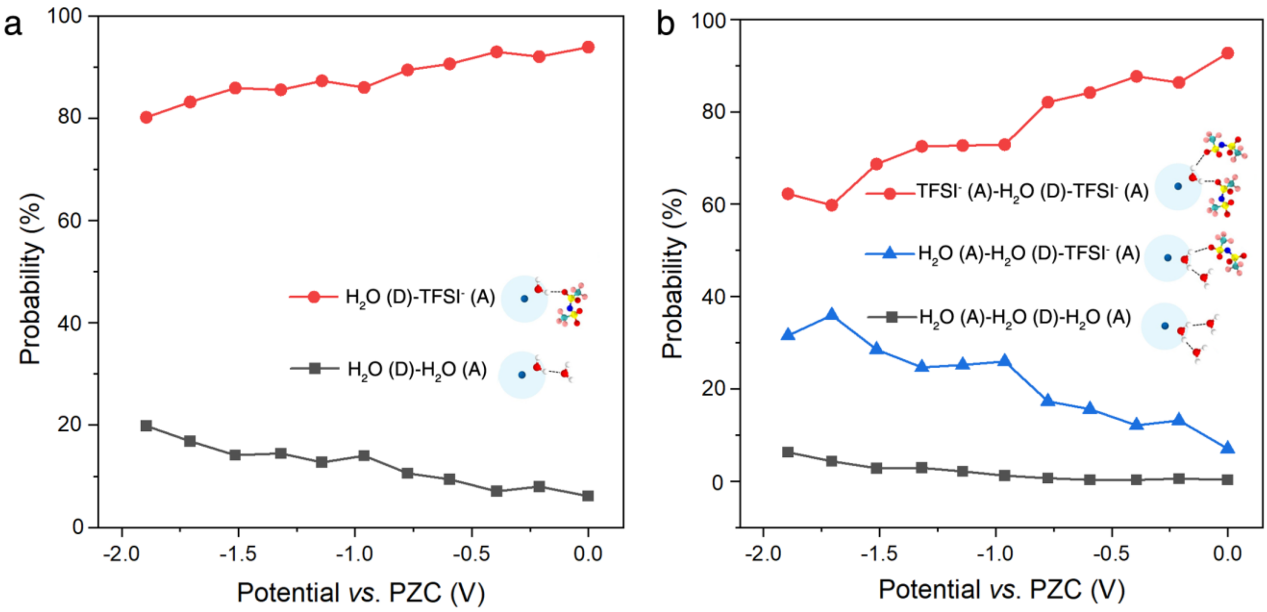

Supplementary Fig. 15 | a-b, Simulated potential-dependent probabilities (%) of Li^+-bound interfacial water with (**a**) one H-bond donor and (**b**) two H-bond donors in 21 m LiTFSI aqueous electrolyte. In the formation of H-bond with a Li^+-bound water as donor (D), another water molecule or $TFSI^-$ can act as an acceptor (A), e.g., for interfacial water with one H-bond donor: H_2O (D)-$TFSI^-$ (A) and H_2O (D)-H_2O (A); for interfacial water with two H-bond donors: $TFSI^-$ (A)-H_2O (D)-$TFSI^-$ (A), H_2O (A)-H_2O (D)-$TFSI^-$ (A), and H_2O (A)-H_2O (D)-H_2O (A).

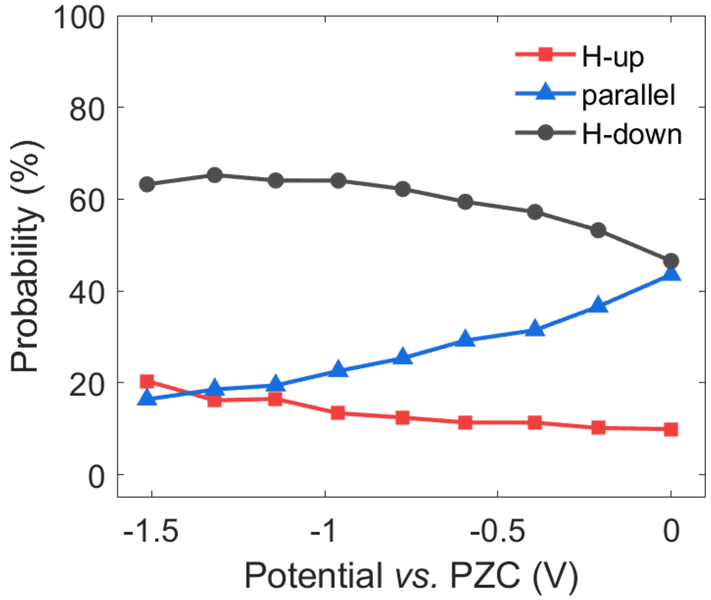

Supplementary Fig. 16 | The percentage of Li^{+}-bound water with different configurations.

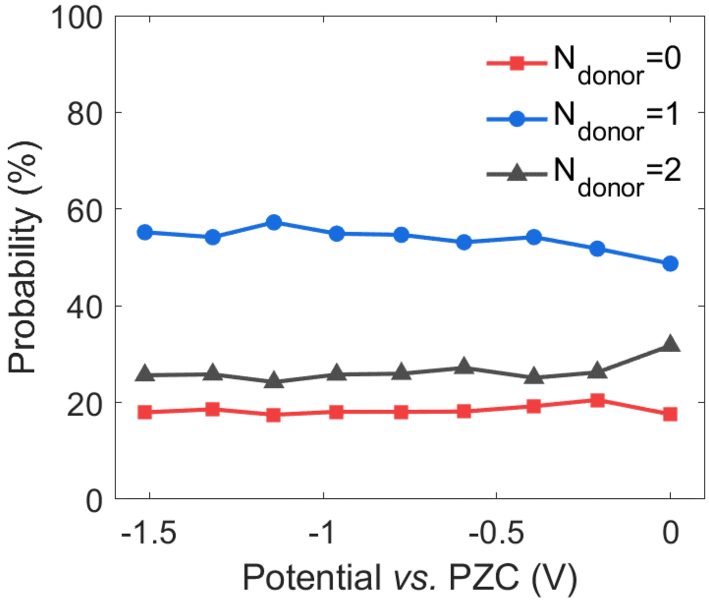

Supplementary Fig. 17 | Potential-dependent probability (%) of interfacial 'H-up' Li^{+}-bound water with different donor numbers.

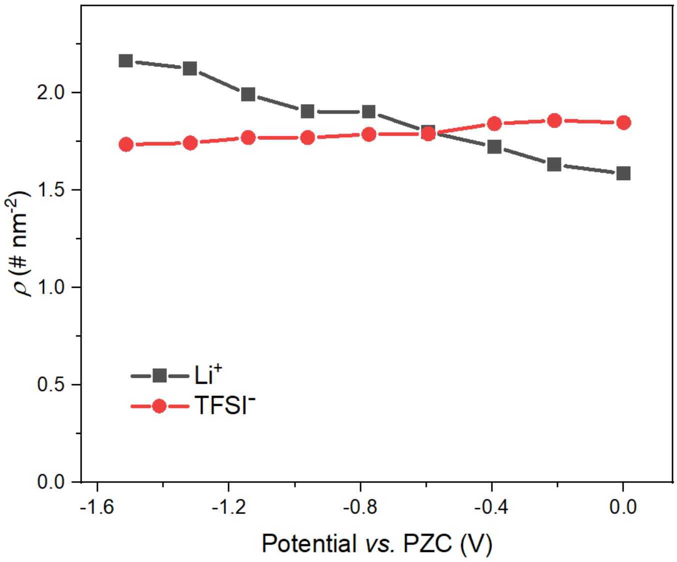

Supplementary Fig. 18 | Simulated potential-dependent accumulative number density of Li^{+} (black square) and $TFSI^{-}$ (red dot) ions in the EDL region.

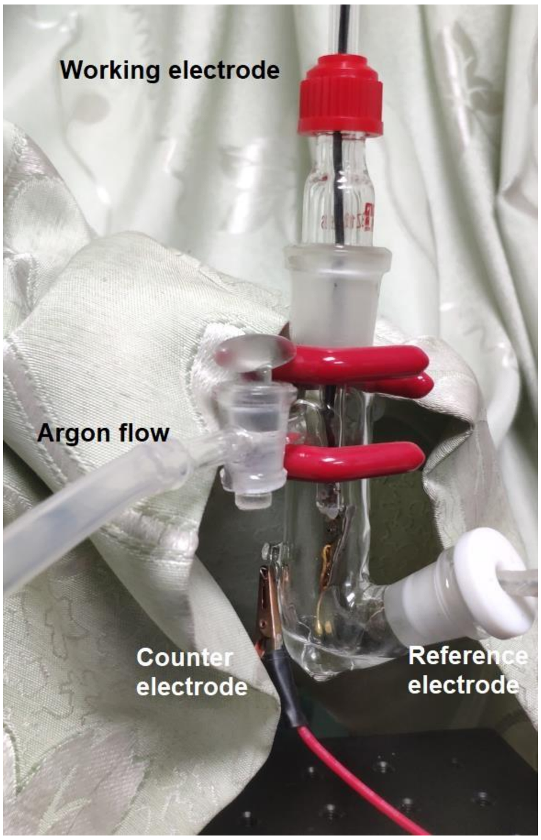

Supplementary Fig. 19 | Photograph of electrochemical cell used for electrochemical measurments.

Supplementary Table 1 | Simulated potential-dependent probability (%) of interfacial Li^{+}-bound water with different donor numbers (*i.e.*, when a water molecule serves as an acceptor or donor, how many H-bonds could be formed).

Probability (%)	Li^{+}-bound water								
	Acceptor = 0			Acceptor = 1			Acceptor = 2		
Potential	Donor = 0	Donor = 1	Donor = 2	Donor = 0	Donor = 1	Donor = 2	Donor = 0	Donor = 1	Donor = 2
0	7.422	42.093	46.824	0.367	2.159	1.128	0.000	0.004	0.002
-0.21	8.966	46.766	39.260	0.599	2.894	1.504	0.002	0.008	0.002
-0.39	9.506	48.009	35.688	0.740	4.357	1.659	0.002	0.037	0.003
-0.59	8.869	48.012	35.305	1.128	5.179	1.476	0.003	0.019	0.008
-0.77	9.360	49.867	30.972	1.178	7.013	1.587	0.002	0.019	0.002
-0.96	9.155	52.200	28.687	1.086	7.187	1.662	0.002	0.018	0.003
-1.14	10.964	53.662	25.121	1.423	7.442	1.364	0.002	0.020	0.003
-1.32	10.503	53.281	24.956	1.389	8.243	1.596	0.003	0.024	0.005
-1.51	11.795	54.311	21.366	1.983	9.081	1.418	0.005	0.036	0.005
-1.71	11.428	53.739	20.573	1.983	10.789	1.413	0.009	0.063	0.004
-1.89	11.933	53.504	20.127	1.931	10.476	1.956	0.008	0.060	0.006

Note: the data marked by red background were used in the interpretation of the interfacial water in Fig. 2.
